# Mentoring to Support Healthcare Professional and Medical Career Progression and Leadership Development

**DOI:** 10.5694/mja2.70169

**Published:** 2026-03-22

**Authors:** Jenny Proimos, Helena J. Teede, Belinda Garth

**Affiliations:** ^1^ Monash Centre for Health Research and Implementation Monash University Melbourne Victoria Australia; ^2^ Monash Health Melbourne Victoria Australia

**Keywords:** health personnel, health systems, professional education, women physicians, women's rights

## Abstract

Mentoring programmes are increasingly used in the health sector to provide career support and guidance for health professionals. However, a number of mentoring experiences and programmes fall short of their potential, with variable outcomes reported. This article summarises the mentoring literature, which clearly demonstrates that mentoring is an important evidence‐informed component of advancing women in leadership. We provide a perspective on mentoring in the context of promoting gender equity within workplaces and propose a new nuanced and integrated model to consider for the advancement of women in leadership.

## 1 Introduction

Mentoring is widely acknowledged as a powerful and effective strategy for career progression, leadership advancement, improved retention, greater influence and meaningful career impact [1, 2]. When done well, it benefits both mentor and mentee—each gaining valuable insights and growing personally and professionally. A mentor's experience and guidance can be especially valuable in helping others navigate the complexities of their career journey. However, while well intentioned, many mentoring programmes fall short, often lacking the structure and evidence‐based approaches needed to achieve their intended outcomes.

## 2 Formal and Informal Mentorship

Mentorship can occur both formally and informally [3]. Formal mentoring is built on a systematic infrastructure that is intentionally established, managed and supported by an organisation to foster staff growth. It typically involves designated mentors and mentees matched through a systematic process, with objectives, timelines and sometimes training or guidelines. Formal programmes can be scaled, evaluated and designed to promote inclusion and equal access—particularly benefiting those who may lack confidence or self‐advocacy skills. However, shortcomings can include a ‘poor fit’ between mentor and mentee resulting in poor personal connection, conflicts around mentors within the same team or organisational context, lack of depth and authenticity, rigidity and enforced time limits that may limit the overall benefits for mentees [3].

Informal mentoring, by contrast, emerges organically—often driven by mutual interest and initiated by the mentee, inspired by role models or shared values. These relationships are founded on strong relational dynamics, which are key to effective mentoring [2]. Benefits include richer developmental experiences, authentic connection built on mutual interest and trust, greater flexibility, personalisation and longevity that can continue beyond formal roles or job changes. However, it risks reinforcing existing inequalities and favouring those with greater social capital or confidence to seek out such connections, who tend to align in gender, race or ethnicity with their mentors [4]. It may also be harder to monitor and align to organisational goals.

Research on the benefits of both informal and formal mentoring vary, with some studies showing greater career support and progression with informal mentoring, compared with formal mentoring [5]. Conversely, other studies show the benefit and return on investment of formal mentoring programmes [6]. Different definitions of mentoring in the literature make it difficult to directly compare formal and informal mentoring. What is clear is that both mentoring models rely on the strength and quality of the relationship between mentor and mentee for their success [7].

## 3 The Mentor–Mentee Relationship

The importance of a strong relationship, trust and impartial guidance makes it vital that mentorship is not entangled with supervisory responsibilities or vested interests. Mentorship can be conflated with clinical supervision for those working in the healthcare system. Clinical supervision focuses on overseeing the performance of a person within their professional role, while mentorship is based on support and guidance of a mentee in personal and professional development, without assessment of performance, although it may encourage self‐assessment or reflection [8]. There is a potential conflict of interest for anyone in a supervision or management role; therefore, it is important for both mentors and mentees to consider this in the establishment of a mentorship relationship. Ideally, mentors should not have a stake in the work of the mentee or be their line manager [1]. One way to counter this is for the mentee to take ownership of choosing a mentor and cultivating the mentor relationship [9]. When doing this, it is equally important to consider the particular skills and experience a mentor offers. Different mentoring skills may be needed at different stages of career, or for different roles; for example, mentoring in leadership, communication, research or clinical career progression. Mentees should be encouraged to consider the skill set that would benefit them in seeking or choosing a mentor [1].

## 4 Impact of Mentoring on Career Advancement and Leadership

Our research has demonstrated the vital role of mentoring in career advancement into leadership for women, including in healthcare and medicine, with a focus on organisational and systemic approaches [10]. Yet many women doctors report that lack of access to mentorship remains a substantial barrier to their career and leadership development [11, 12]. This is important because despite comprising about 75% of the Australian healthcare workforce, women remain under‐represented in leadership roles [13, 14]. For example, in private health services, women hold only 46% of board positions and 29% of board chair roles [15]. Addressing this imbalance aligns with global priorities, including the United Nations' Sustainable Development Goals, the World Health Organization's calls for gender equity in health systems, and the *Lancet* Commission's recommendations on women and health. Evidence shows that advancing women into healthcare leadership improves quality of care, reduces patient mortality rates and leads to more equitable health outcomes—particularly for women and children [16, 17, 18, 19, 20]. Diverse leadership also enhances economic growth, workforce engagement, organisational performance, productivity, profitability and the career trajectories of other women [21].

## 5 Addressing the Healthcare Leadership Gap

To help address the gap in healthcare leadership experienced by women, we have established the Partnership Centre for Gender Equality and Leadership Advancement and the Advancing Women in Healthcare Leadership (AWHL) national initiative [22, 23], supported by National Health and Medical Research Council partnership funding, in collaboration with 28 partners. These partners represent private and public health services, professional colleges, industrial bodies, government and women in the healthcare workforce. This implementation research and translation initiative seeks to implement multi‐level evidence‐based strategies to support women's leadership progression. Given the powerful role of mentoring on career and leadership development [7], ensuring that equitable access to mentoring is a key objective of AWHL.

Feedback from participants in our leadership development programmes over more than a decade has shown that women—particularly early in their careers—are reluctant to seek out mentors due to structural, relational and cultural barriers. Gendered norms, self‐perception and social conditioning often discourage self‐advocacy and assertiveness. A reluctance to impose on others, shaped by confidence and self‐worth, further limits women's engagement in mentoring relationships [11, 24]. These dynamics can limit access to opportunities that are important for career and leadership development, including mentoring, especially for individuals who experience overlapping and systemic barriers at the intersection of gender, race, sexuality, ethnicity and disability [25, 26].

**FIGURE 1:**
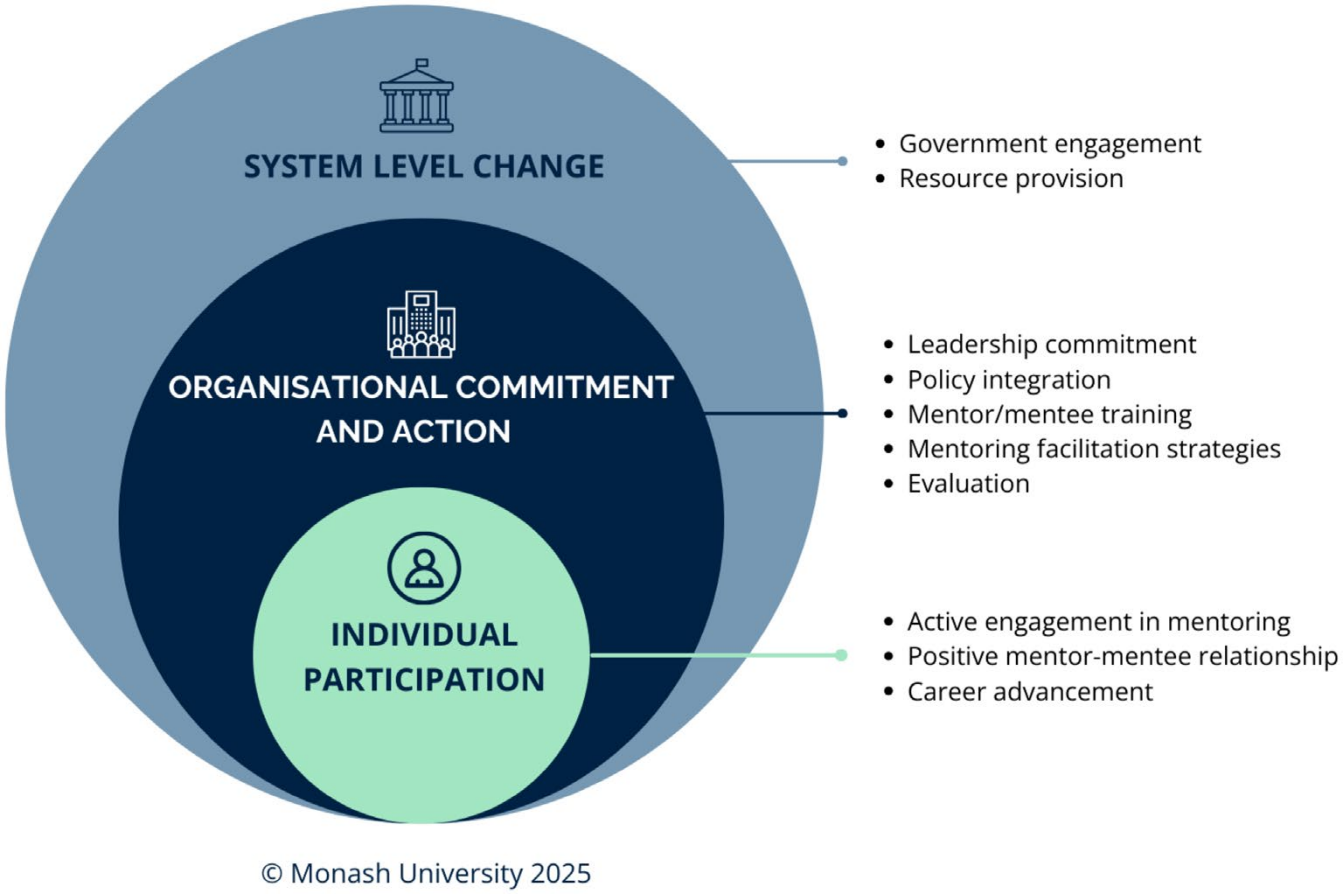
Proposed mentoring ecosystem that is adaptive, inclusive and responsive to diverse healthcare workforce needs. Figure reproduced with permission from Monash University.

In this context, and based on evidence from across sectors, including healthcare, we argue that there is a compelling opportunity to codesign and implement effective systemic mentoring approaches at national, systems and organisational levels that encompass both formal and informal approaches. These should be adaptive, inclusive and responsive to diverse needs and tailored to the sector (Figure 1). This builds on the importance of intentionality, equity and adaptability in mentoring design, emphasising that effective mentoring requires both organisational or systemic structural support and individual authentic, relational connection.

## 6 Next Steps

To optimise equitable access to effective mentoring and advance gender equity in healthcare leadership, we propose a nuanced, integrated and evidence‐informed mentoring model. This approach combines the structure and inclusivity of formal mentoring with the relational depth of informal connections. It requires systems‐level support, including policy engagement and incentive strategies (e.g., access to credits for continuous professional development) and broad provision of quality, evidence‐based resources and training.

Within healthcare and medical organisations, this can be operationalised through leadership commitment and role modelling, embedding mentoring into position descriptions and performance reviews, providing accessible mentee and mentor training, facilitating self‐matching strategies such as networking and ‘speed matching’ events. For healthcare organisations employing the healthcare workforce, integrating internal organisational formal mentoring to optimise equality, with built‐in evaluation, is also vital. Crucially, fostering a culture that encourages, normalises and expects the development of intentional and effective mentor–mentee relationships is essential, followed by evaluation of their impact (Table 1).

**TABLE 1:** A multilevel model of mentoring.

System level actions
Government engagement (e.g., support for non‐clinical time) College inclusion in continuing professional development frameworks Resource provision (e.g., College development of mentorship training and education)
Organisation level actions
Leadership commitment Integration into organisational policies and procedures Provision of training for mentors and mentees Mentoring facilitation strategies (e.g., non‐clinical time, networking events) Evaluation of mentoring strategies
Individual level actions
Active engagement in mentoring (both as mentor and mentee) Positive mentor–mentee relationship (allow for flexibility to find the right mentor) Mentor support for career advancement

We are currently working with our AWHL partners across all levels of the healthcare system to implement these evidence‐based approaches to mentoring. This includes the federal Australian Medical Association, which is advocating for and implementing evidence‐based mentoring strategies at a national scale. In this context, we encourage organisations—including health services, professional colleges and universities—to adopt a broader, evidence‐informed approach to implementing mentoring programmes as a strategic lever for advancing gender equity in healthcare leadership.

## Author Contributions

**Jenny Proimos:** conceptualisation, project administration, writing draft, writing (review and editing). **Helena J. Teede:** conceptualisation, writing (review and editing). **Belinda Garth:** conceptualisation, writing (review and editing).

## Funding

This article is one of the outputs of the Advancing Women in Healthcare Leadership national initiative, funded by National Health and Medical Research Council (NHMRC) Partnership Grants (APP1198561 and 2018718). NHMRC has had no role in the design or drafting of this article. Helena Teede receives funding from an NHMRC Fellowship.

## Disclosure

The authors have nothing to report.

## Conflicts of Interest

The authors declare no conflicts of interest.

## Data Availability

The authors have nothing to report.
